# Tumor‐driven like macrophages induced by conditioned media from pancreatic ductal adenocarcinoma promote tumor metastasis via secreting IL‐8

**DOI:** 10.1002/cam4.1824

**Published:** 2018-10-12

**Authors:** Shao‐jie Chen, Guo‐da Lian, Jia‐jia Li, Qiu‐bo Zhang, Lin‐juan Zeng, Ke‐ge Yang, Chu‐mei Huang, Ya‐qing Li, Yin‐ting Chen, Kai‐hong Huang

**Affiliations:** ^1^ Department of Gastroenterology, Guangdong Provincial Key Laboratory of Malignant Tumor Epigenetics and Gene Regulation Sun Yat‐sen Memorial Hospital, Sun Yat‐sen University Guangzhou China; ^2^ Department of Nephrology Sun Yat‐sen Memorial Hospital, Sun Yat‐sen University Guangzhou China; ^3^ Department of Gastroenterology Lihuili Hospital of Ningbo Medical Center Ningbo China; ^4^ Department of Oncology The Fifth Affiliated Hospital of Sun Yat‐sen University Zhuhai China

**Keywords:** epithelial‐mesenchymal transition, IL‐8, inflammatory microenvironment, pancreatic ductal adenocarcinoma, tumor‐associated macrophages

## Abstract

Tumor‐associated macrophages (TAMs) are abundant population of inflammatory cells which play an essential role in remodeling tumor microenvironment and tumor progression. Previously, we found the high density of TAMs was correlated with lymph node metastasis and poor prognosis in pancreatic ductal adenocarcinoma (PDAC). Therefore, this study was designed to investigate the mechanisms of interaction between TAMs and PDAC. THP‐1 monocytes were the exposure to conditioned media (CM) produced by PDAC cells; then, monocyte recruitment and macrophage differentiation were assessed. CM from PDAC attracted and polarized THP‐1 monocytes to tumor‐driven like macrophages. mRNA expression cytokine profiling and ELISA identified the IL‐8 secretion was increasing in tumor‐driven like macrophages, and STAT3 pathway was involved. Addition of exogenous recombinant human IL‐8 promoted PDAC cells motility in vitro and metastasis in vivo via upregulating Twist expression, which mediated epithelial‐mesenchymal transition in cancer cells. What is more, IL‐8 expression level in tumor stroma by immunohistochemical analysis was related to lymph node metastasis, the number of tumor CD68 but not CD163 positive macrophages and patient outcome. Taken together, these findings shed light on the important interplay between cancer cells and TAMs in tumor microenvironment and suggested that IL‐8 signaling might be a potential therapeutic target for PDAC.

## 1 INTRODUCTION

Pancreatic ductal adenocarcinoma (PDAC) is the fourth leading cause of cancer‐related death in Western societies with an overall 5‐year survival rate of approximately 3%‐5% and the median survival of less than 6 months.^1^ Continuously, accumulating evidence reveals that tumor inflammatory microenvironment plays an essential role in the development and progression of PDAC, which may provide novel therapeutic approaches for the treatment,^2^ whereas the interaction between inflammatory microenvironment and PDAC cells is poorly understood.

Macrophages are the most abundant cells resident in the tumor microenvironment, referred to as tumor‐associated macrophages (TAMs) and have great influence on tumor progression.^3^ TAMs preferentially polarize toward a M2‐like phenotype (alternatively activated macrophages) due to the absence of M1‐orienting signals as well as to the expression of M2 stimuli in the tumor, which exert pro‐tumor effects.^4^ Clinical and epidemiological evidence have demonstrated a significant association between TAMs density and poor prognosis in various cancers, including PDAC.^5^, ^6^, ^7^ In our previous study, we found that increased density of TAMs was correlated with lymph node metastasis and decreased patients’ survival in PDAC.^8^ It is reported that coculture PDAC cells with TAMs derived from in vitro cell models or tumor tissue promotes cancer cell metastasis via epithelial‐mesenchymal transition (EMT) process.^9^, ^10^ Simultaneously, supernatant produced by PDAC cells is able to polarize human peripheral blood mononuclear cells (PBMCs) or monocyte cell lines into TAMs which display a highly invasive signature.^11^, ^12^ However, it is not fully elucidated how the crosstalk between cancer cells and TAMs facilitates PDAC development and progression.

In the present study, we first focused our work on the recruitment and differentiation of monocytes by stimulation from cancer cells. To mimic a tumor microenvironment in vitro, THP‐1 monocytes were attracted and exposed to conditioned media (CM) of PDAC cells and differentiated into tumor‐driven like macrophages. THP‐1 monocytes are an appropriate alternative to PBMCs due to the high growth and the absence of donor‐related variability, and they retain many properties of native monocyte‐derived macrophages.^13^ Next, we identified the cytokine/chemokines produced by the above tumor‐driven like macrophages which had pro‐tumor effect. Furthermore, the pro‐tumor molecular mechanism was also investigated. At last, we provided evidence that PDAC cells recruited and polarized THP‐1 monocytes toward tumor‐driven like macrophages in tumor microenvironment where they accelerated cancer metastasis.

## 2 MATERIALS AND METHODS

### 2.1 Materials

IL‐8 was purchased from Pepro Tech Inc (Rocky Hill, NJ), and IL‐8 ELISA Kit was purchased from R&D Systems (Minneapolis, MN). SiRNA oligos targeted Twist gene was purchased from GenePharma (Shanghai, China). Antibodies used in Western blotting were listed in Appendix S1.

### 2.2 Cell culture

The hTERT‐immortalized human pancreas nestin expressing (hTERT‐HPNE) cell lines, the human pancreatic adenocarcinoma cell lines PANC‐1, BxPC‐3, and human leukemia monocyte THP‐1 cell lines all were purchased from American Type Culture Collection (Rockefeller, MD). hTERT‐HPNE cells were maintained in M3:5 growth medium as previously described.^14^ While PANC‐1, BxPC‐3, and THP‐1 cells were cultured in DMEM or RPMI 1640, respectively, supplemented with 10% fetal bovine serum (Biological Industries, Beit Haemek, Israel), all cells were maintained in a humified 5% CO_2_ atmosphere at 37°C.

### 2.3 Preparation of conditioned media

hTERT‐HPNE, PANC‐1, and BxPC‐3 cells were grown to 80% of confluence, washed twice, and changed to RPMI 1640 without fetal bovine serum. CM was collected and filtered at 0.20 µm after 48 hours incubation, and the supernatant was stored at −80°C until further use. In STAT3 pathway analysis, THP‐1 cells were treated with 15 µmol/L STATTIC (Calbiochem, San Diego, CA) for 1 hour and corresponding 50% v/v CM of PDAC cells for 48 hours, then washed with PBS, and incubated with fresh RPMI 1640 for 24 hours.

### 2.4 Monocyte chemotaxis and adhesion assay

Chemotaxis assays were conducted in 24‐well modified Boyden chamber (Corning Life Sciences, Corning, MA) as previously described,^15^ and adhesion assays were performed in 96‐well plate coated with 40 µg/mL Matrigel (BD Biosciences, Bedford, MA) (details in Appendix S1).

### 2.5 Immunofluorescent staining experiments

After treating with 50% v/v PANC‐1‐CM or BxPC‐3‐CM for 48 hours, THP‐1 monocytes became adhesion, aggregation and were used for immunofluorescence double staining (coexpression) of CD68 and CD163. The cells were fixed with methanol for 15 minutes at room temperature. Subsequently, the cells were incubated with primary antibodies against CD68 (1:150; Dako, Glostrup, Denmark) and CD163 (1:150; Abcam, Cambridge, UK) at 4°C overnight. After incubation with Alexa Fluor 488‐conjugated secondary antibody (1:200; Invitrogen, Carlsbad, CA) and Alexa Fluor 647‐conjugated secondary antibody (1:200; Invitrogen) at room temperature in the dark for 2 hours, the cells were incubated with DAPI for 5 minutes. Immunoreactivity signals were viewed under confocal microscope (LSM 710; Carl Zeiss, Oberkochen, Germany).

### 2.6 Transwell migration and invasion assays

Cell migration and invasion was estimated using 24‐well transwell chambers polycarbonate filter of 8 µm pore size (Corning Life Sciences). For invasion assay, chambers were pre‐coated with 250 µg/mL Matrigel to the upper surface of each filter. The experiment procedure was described in Appendix S1.

### 2.7 PDAC tissue collection and immunohistochemistry

A total of 70 cases of PDAC patients who underwent a primary resection at Sun Yat‐sen Memorial Hospital of Sun Yat‐sen University (Guangzhou, China) from September 2004 to December 2011 were enrolled in the study. In addition, tissue from 10 patients who underwent pancreas surgery because of injuries was also collected as control. Clinical materials were used for research purposes after the Research Ethics Committee of Sun Yat‐sen Memorial Hospital approved the study.

The immunohistochemical stain was performed as we previously described^8^, ^16^ using anti‐IL‐8 antibody (1:100; Santa Cruz Biotechnology, Santa Cruz, CA). The degree of immunostaining of the sections was viewed and calculated, respectively, by two independent investigators who were blinded to the clinicopathologic data using the method which Zhang et al^17^ have reported before. Thus, the results divided into two groups (namely the low IL‐8 expression group and the high IL‐8 expression group) and went for clinical analysis.

### 2.8 Animal study

All animal experiments were approved by both the National Institutes of Health Guide for the Care and Use of Laboratory Animal and the Animal Care and Use Committee of Sun Yat‐sen University. Female BALB/c (nu/nu) mice of ages 4‐5 weeks were purchased from the animal facility of Sun Yat‐sen University (Guangzhou, China). To evaluate the effects of IL‐8 on metastatic potential, a single‐cell suspension of PANC‐1 cells (2 × 10^6^ cells) in 0.2 mL of PBS was injected into tail vein on day 0, and exogenous recombinant human IL‐8 (1 μg per mouse and PBS as control) was injected via peritoneal cavity from −1 to day 3. On the day of injection, IL‐8 was injected 2 hours before pancreatic cancer cells, and the subsequent doses were given in intervals of 24 hours.^18^ On day 21, the mice were sacrificed and dissected. Both the livers and the lungs were collected and fixed with 4% paraformaldehyde. H&E staining and immunohistochemical staining were performed according to the manufacturer's protocols.

### 2.9 Statistical analysis

Statistical analyses were carried out using SPSS 13.0 (SPSS Inc., Chicago, IL). Data were presented as mean ± SD. Statistical significance for comparisons between groups was analyzed using Student's *t* test, and one‐way ANOVA test was used for multiple comparisons. Chi‐square test was used to evaluate the difference between IL‐8 expression and the clinicopathologic characteristics. Survival curves were plotted by the Kaplan‐Meier method and compared by the log‐rank test. All the data were obtained from three independent experiments. *P* values <0.05 were considered to be statistically significant in all cases.

## 3 RESULTS

### 3.1 CM of PDAC cells promoted THP‐1 monocytes migration and adhesion

**Figure 1:**
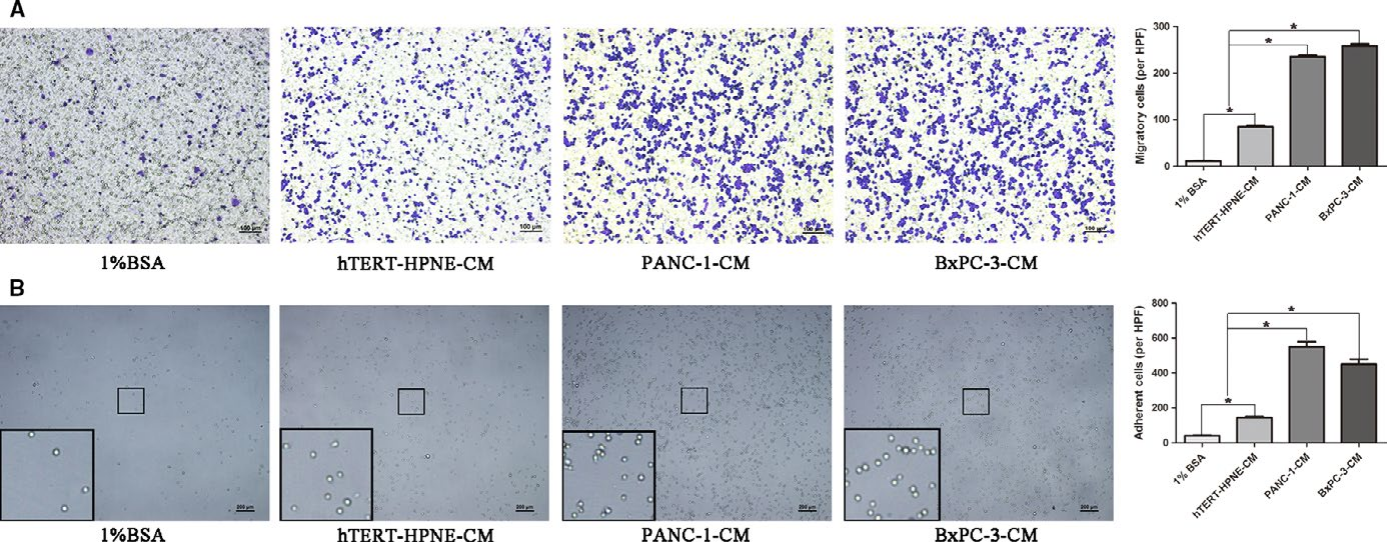
Conditioned media (CM) produced by pancreatic ductal adenocarcinoma cells promoted THP‐1 monocytes migration and adhesion. A, Extent of THP‐1 monocytes migration was evaluated by 24‐well modified Boyden chamber. Briefly, 100 µL of THP‐1 monocytes suspension (1 × 10^6^/mL) was loaded into the insert of Boyden chamber, and the lower well contained either control media (1% BSA) or indicating CM (hTERT‐HPNE‐CM, PANC‐1‐CM or BxPC‐3‐CM). After allowing migrating for 1 h at 37°C in an atmosphere of 5% CO_2_, migrated THP‐1 monocytes were fixed with methanol and stained with Crystal Violet dye. B, THP‐1 monocytes (1 × 10^5^/100 µL) suspended in indicating CM (1% BSA, hTERT‐HPNE‐CM, PANC‐1‐CM, or BxPC‐3‐CM) were added to 96‐well plate pre‐coated with 40 µg/mL Matrigel. After incubating at 37°C in an atmosphere of 5% CO_2_ for 1 h to adhere, the non‐adhered cells were removed by gentle washing PBS for three times. 1% BSA was used as negative control, and quantitative analysis was performed with histogram. Magnification: 100×; Scale bars: 100 μm. *P < 0.05

It is a critical first step that circulating monocytes migrate into the tumor site and differentiate into TAMs which may interact with tumor cells and are involving in shaping tumor microenvironment. Therefore, we explored the recruitment of THP‐1 monocytes in response to CM produced by PANC‐1 and BxPC‐3 cell lines. THP‐1 monocytes exhibited greater migration toward CM from cancer cells (PANC‐1‐CM and BxPC‐3‐CM) than that from normal pancreatic duct epithelial cells (hTERT‐HPNE‐CM), and 1% BSA was used as negative control in which few cells migrated into the membrane. Quantitative analysis showed the number of THP‐1 monocytes that was attracted by PANC‐1‐CM or BxPC‐3‐CM was over twofold increase than that of hTERT‐HPNE‐CM did (Figure 1A). Next, we examined the effect of collected CM on adhesion of THP‐1 monocytes to Matrigel and obtained the similar results as chemotaxis assay, which showed that the number of adherent THP‐1 monocytes from PANC‐1‐CM or BxPC‐3‐CM went nearly three times higher than that of hTERT‐HPNE‐CM (Figure 1B). These results suggested that THP‐1 monocytes acquired the enhanced ability of migration and adhesion in respond to CM produced by PANC‐1 and BxPC‐3 cell lines.

### 3.2 CM of PDAC cells induced THP‐1 monocytes to polarize toward tumor‐driven like macrophages

**Figure 2:**
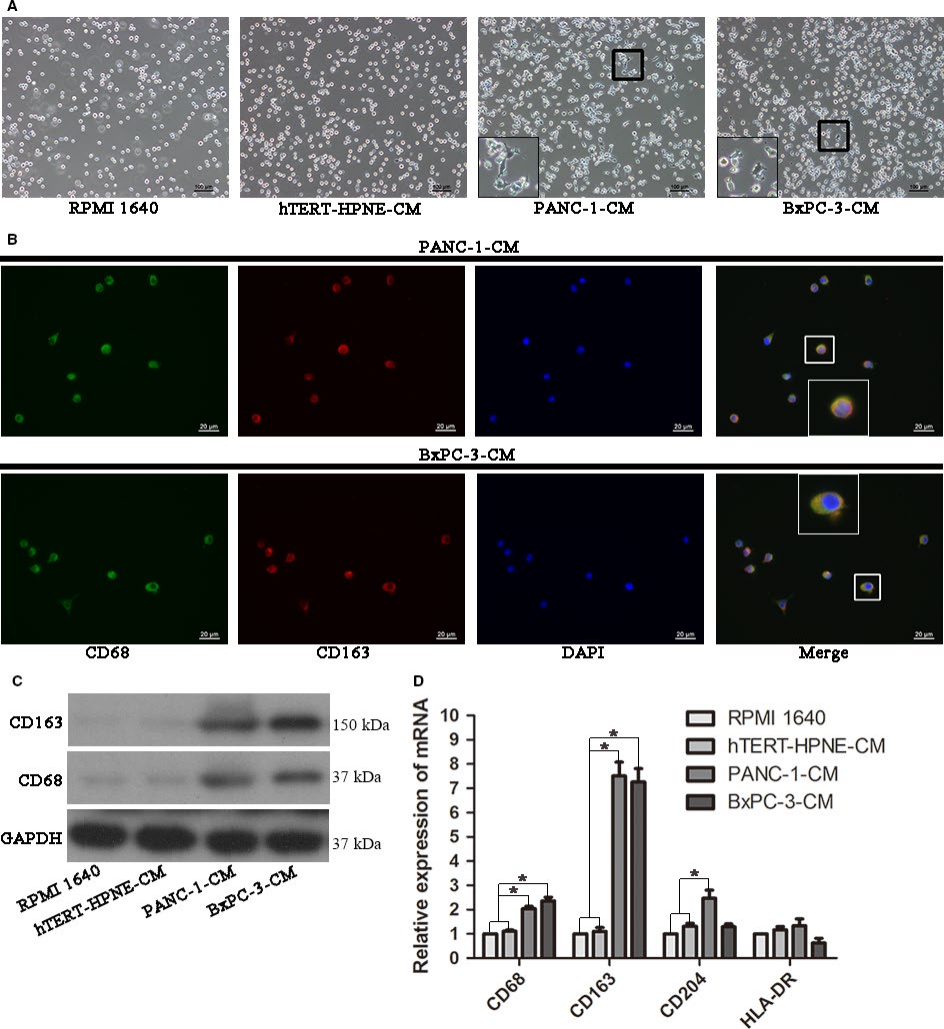
Effect of conditioned media (CM) produced by pancreatic ductal adenocarcinoma cells on THP‐1 monocytes differentiation. A, Representative images of cells stimulated by corresponding CM for 48 h. Magnification: 100×; Scale bars: 100 μm. B, THP‐1 monocytes were treated with 50% v/v PANC‐1‐CM or BxPC‐3‐CM for 48 h to promote cell differentiation before immunofluorescent staining. Immunofluorescence detection of CD68 (green) and CD163 (red) demonstrated their coexpression in tumor‐driven like macrophages. Magnification: 400×; Scale bars: 20 μm. C, The expression of CD68 and CD163 in different CM‐treated THP‐1 cells was detected by Western blotting. D, The mRNA expression of CD68, CD163, CD204, and HLA‐DR was examined by qRT‐PCR. *P < 0.05

It has been reported in several studies that culture supernatants from cancer cells can promote PBMCs differentiate into TAMs.^11^, ^12^, ^19^ Thus, we would like to figure out whether CM from PDAC cells has the similar effect on THP‐1 monocytes following their recruitment to the tumor site. THP‐1 monocytes were exposed to CM of PANC‐1 or BxPC‐3. After incubation in CM of PDAC cells for 24 hours, we observed that THP‐1 monocytes became attached to the bottom of culture plate, evident morphological modifications, and more importantly, pseudopods were noticed in some cells (Figure 2A). However, hTERT‐HPNE‐CM did not affect THP‐1 monocytes apparently compared with the RPMI 1640 control. To further verify these cells had differentiated into TAMs, they were performed immunofluorescence double staining of CD68 and CD163, in which both were the most common used markers of TAMs. As shown in Figure 2B, THP‐1 derived macrophages that stimulated with PDAC cells’ CM were coexpressed CD68 and CD163, which were complete overlap staining, and the result was further confirmed by Western blotting (Figure 2C). So, we named this kind of TAMs as tumor‐driven like macrophages throughout the text. Then, we also did qRT‐PCR analysis of CD68, CD163, CD204, and HLA‐DR to verify their polarization. The data demonstrated that mRNA expression level of CD68 (marker of macrophages), CD163, and CD204 (markers of M2 phenotype macrophages) were increased compared to hTERT‐HPNE‐CM and RPMI 1640 negative control, while HLA‐DR (marker of M1 phenotype macrophages) did not obviously decreased (Figure 2D). This might suggest tumor‐driven like macrophages displayed a M2‐like phenotype polarization. Taken together, these results indicated that CM produced by PDAC cells but not normal pancreatic ductal epithelial cells could recruit THP‐1 monocytes and polarize them toward tumor‐driven like macrophages.

### 3.3 Tumor‐driven like macrophages secreted IL‐8 through STAT3 pathway to promote PDAC cells motility

**Figure 3:**
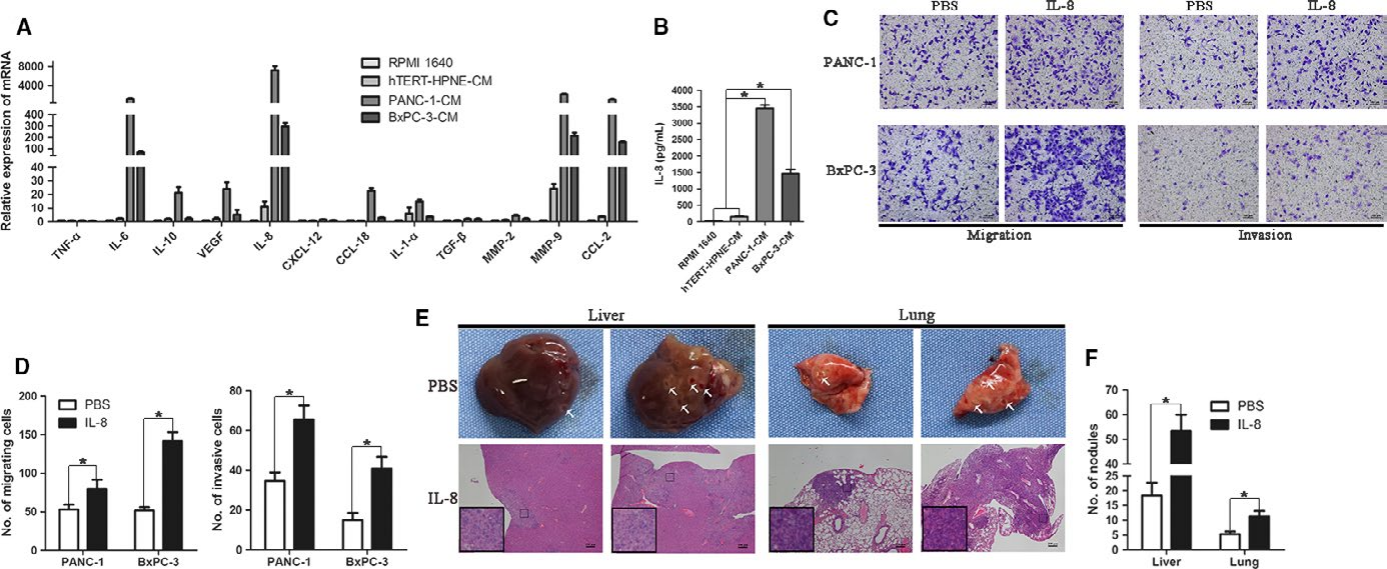
Tumor‐driven like macrophages secreted IL‐8 to enhance pancreatic ductal adenocarcinoma cells motility. A, THP‐1 monocytes were treated with different conditioned media (CM; RPMI 1640, hTERT‐HPNE‐CM, PANC‐1‐CM, or BxPC‐3‐CM) for 48 h. The RNAs was extracted from different cells by Trizol reagent, and then, cDNA was synthesized.The mRNA expression of cytokines in different CM‐treated THP‐1 cells was assayed by qRT‐PCR. B, IL‐8 protein expression level in supernatant of different CM‐treated THP‐1 cells was determined by ELISA. C, PANC‐1 and BxPC‐3 cells (2 × 10^4^/well) treated with 100 ng/mL IL‐8 or PBS were evaluated their migratory and invasive abilities by Transwell assay. Magnification: 100×; Scale bars: 100 μm. D, Histogram of average numbers of penetrated cells per microscopic field. E, A single‐cell suspension of PANC‐1 cells (2 × 10^6^ cells) in 0.2 mL of PBS was injected into tail vein of nude mice, and exogenous recombinant human IL‐8 (1 μg per mouse and PBS as control) was injected via peritoneal cavity. On day 21, the mice were sacrificed and dissected. Gross metastatic lesion in liver and lung and their H&E staining were showed in the images. Magnification: 40×; Scale bars: 200 μm. F, Graphic representation of liver and lung metastatic lesions per slide under microscopy. *P < 0.05

To confirm the expression change of cytokines in tumor‐driven like macrophages, we performed qRT‐PCR to screen a panel of cytokines. Elevated levels of IL‐6, IL‐8, MMP‐9, and CCL‐2 were observed. Of note, IL‐8 level was the most abundantly expressed cytokine both in PANC‐1‐CM and BxPC‐3‐CM (Figure 3A). In addition, hTERT‐HPNE‐CM also had slight stimulation in cytokines secretion, but the effect was far weaker than PDAC cells. To verify IL‐8 protein expression level, we examined supernatant from the above cells using ELISA Kit, and the results were in consistent with qRT‐PCR analysis (Figure 3B). Subsequently, we investigated what influence IL‐8 had on PDAC cells. For migration and invasion, IL‐8 significantly enhanced the migrating and invasive ability of PDAC cells in vitro (Figure 3C,D). Moreover, IL‐8 induced more metastatic lesions in the liver and lung than PBS in nude mice injected with PANC‐1 cells via tail vein (Figure 3E). The average number of metastatic nodules increased nearly threefold for mice treated with IL‐8 than PBS (Figure 3F). In addition, we also carried out colony formation and MTT assays but did not acquire a significant change of cell proliferation (Figure S1).

**Figure 4:**
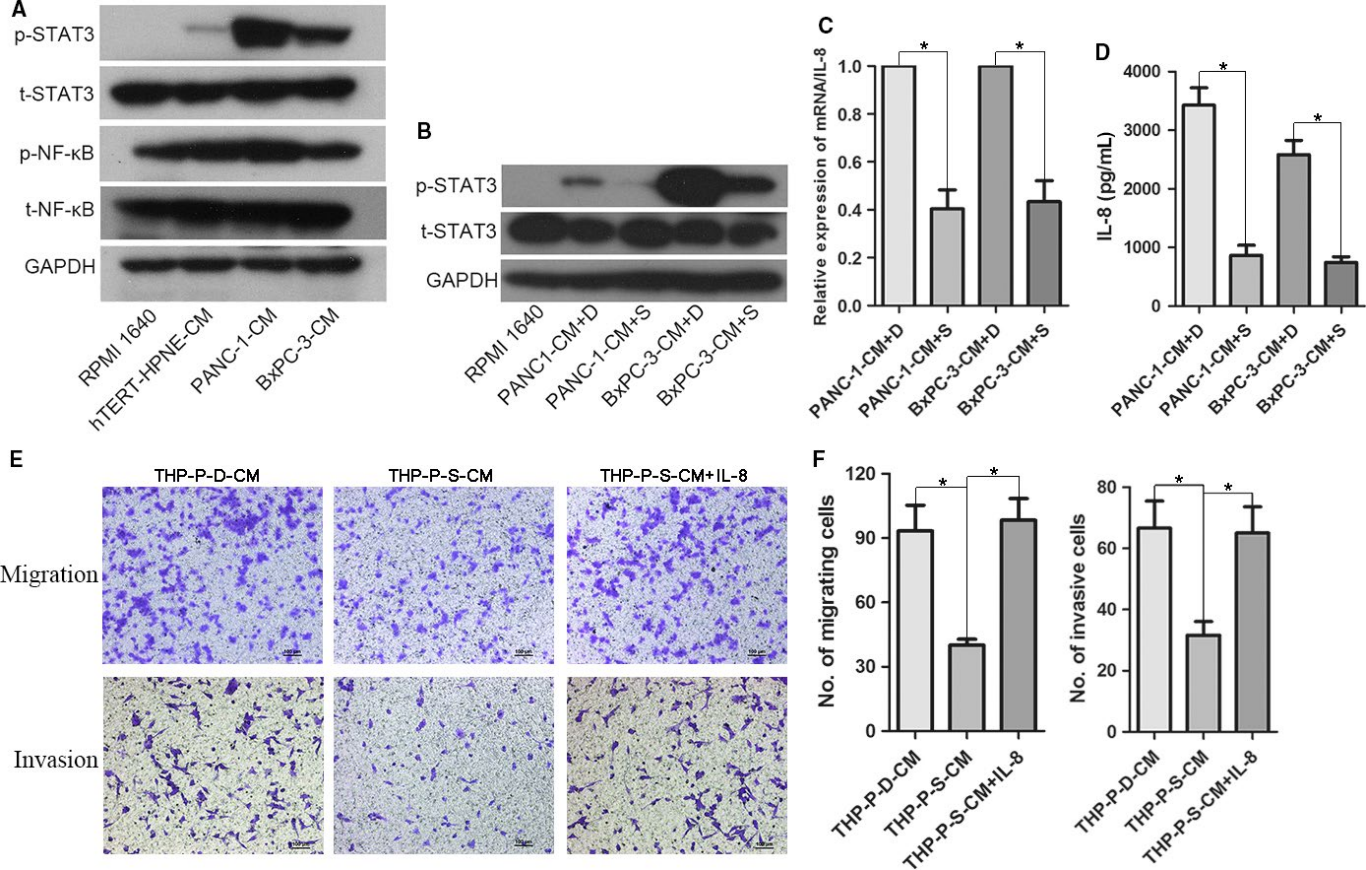
Tumor‐driven like macrophages secreted IL‐8 through activating STAT3 pathway. A, THP‐1 monocytes were cultured with corresponding conditioned media (CM) for 48 h and analyzed for levels of phosphorylated STAT3 (p‐STAT3) and NF‐κB (p‐NF‐κB). B, The effective inhibition of p‐STAT3 by STATTIC was confirmed by Western blotting. C, The mRNA expression level of IL‐8 in tumor‐driven like macrophages at the presence of DMSO or STATTIC (15 µmol/L, dissolved in DMSO) was evaluated by qRT‐PCR. D, IL‐8 protein expression level in tumor‐driven like macrophages at the presence of DMSO or STATTIC (15 µmol/L) was evaluated by ELISA. E, THP‐1 monocytes were treated with 15 µmol/L STATTIC for 1 h and corresponding 50% v/v CM of PANC‐1 cells for 48 h, and then, the CM was collected. Migratory and invasive abilities of PANC‐1 under different conditions were determined by Transwell assay. F, Histogram of average numbers of penetrated cells per microscopic field. Magnification: 100×; Scale bars: 100 μm. *P < 0.05. THP‐P‐D‐CM: CM produced by THP‐1 cells cultured in the presence of 50% v/v PANC‐1‐CM and DMSO; THP‐P‐S‐CM: CM produced by THP‐1 cells cultured in the presence of 50% v/v PANC‐1‐CM and STATTIC

We next explored the mechanism by which tumor‐driven like macrophages secreted IL‐8. To our best knowledge, NF‐κB and STAT3 pathways play important roles in control of communication between cancer cells and inflammatory cells,^20^ and furthermore, both of them are involved in regulating the secretion of IL‐8.^21^, ^22^ Therefore, we detected whether the two pathways were activated in tumor‐driven like macrophages via Western blotting after exposing to corresponding CM for 48 hours. We observed that there was significant upregulation of phosphorylated STAT3 (pSTAT3) in tumor‐driven like macrophages than hTERT‐HPNE‐CM‐treated or parent THP‐1 monocytes, and we also found that hTERT‐HPNE‐CM upregulated pSTAT3 level in THP‐1 monocytes slightly (Figure 4A). These results were parallel to the mRNA expression level of cytokines. However, phosphorylated NF‐κB protein level did not change obviously. In an effort to identify that tumor‐driven like macrophages secreted IL‐8 in a STAT3‐dependent mechanism, we examined the mRNA and protein expression level of IL‐8 followed by using STATTIC, a small molecule selective inhibitor of STAT3 pathway. The efficient inhibition of STATTIC on activation of STAT3 pathway by tumor CM was testified before the experiment (Figure 4B), and the experiment data showed that STATTIC‐treated tumor‐driven like macrophages decreased expression of IL‐8 in both mRNA and protein level (Figure 4C,D). In addition, the mobility of PDAC cells decreased more than onefold in comparison with CM treated by STATTIC, while this effect was reversed by adding exogenous IL‐8 (Figure 4E,F). Collectively, these results suggested that tumor‐driven like macrophages secreted IL‐8 via STAT3 pathway to accelerate migration and invasion, not proliferation of PDAC cells.

### 3.4 IL‐8 induced epithelial‐mesenchymal transition in PDAC cells by upregulating the expression of Twist

**Figure 5:**
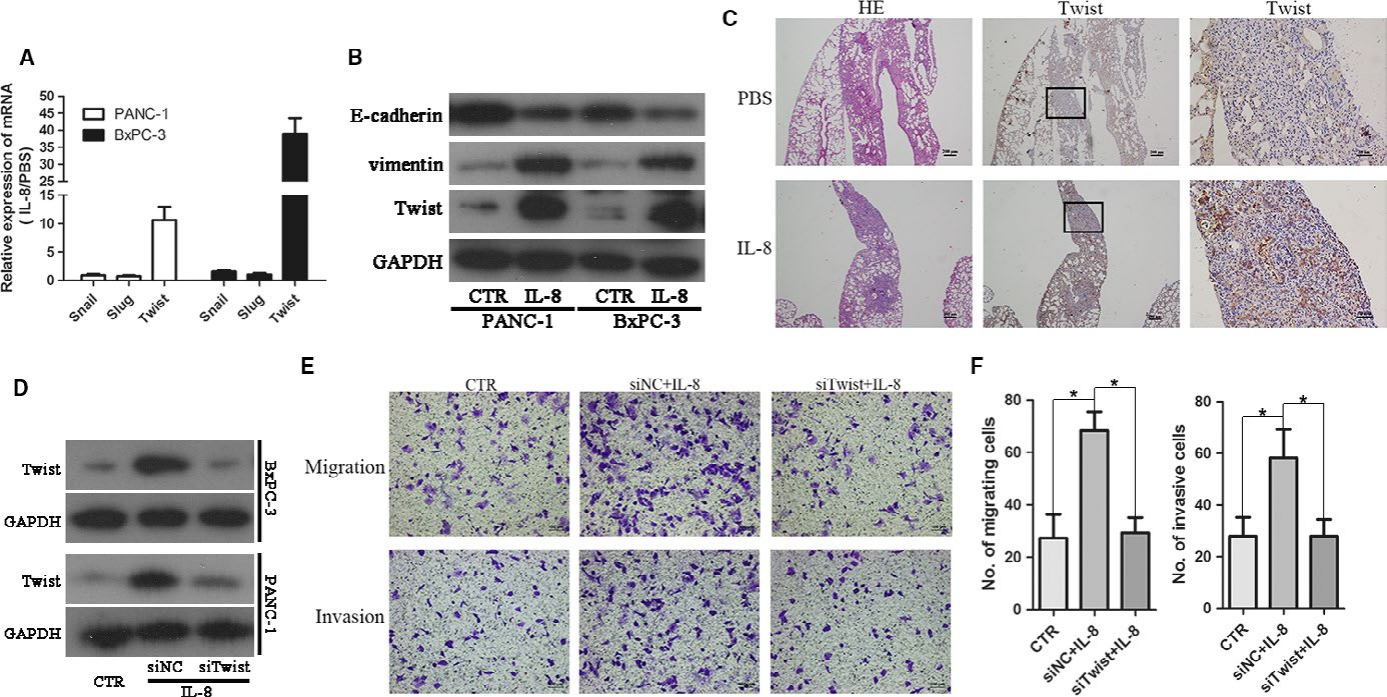
IL‐8 mediated epithelial‐mesenchymal transition (EMT) in pancreatic ductal adenocarcinoma (PDAC) cells via upregulating the expression of Twist. A, qRT‐PCR analysis of EMT regulatory factors including Snail, Slug, and Twist in PANC‐1 and BxPC‐3 cells treated with IL‐8 for 24 h. B, The expression of E‐cadherin, vimentin, and Twist was evaluated by Western blotting in PDAC cells exposure to IL‐8 (100 ng/mL). C, Representative images of histopathological analysis with H&E and immunohistochemical staining of Twist for dissected metastatic lesion tissue from lung. D, Efficiency of Twist knockdown in PDAC cells by siRNA was confirmed by Western blotting. E, The motility ability of PANC‐1 cells was assessed by Transwell assay in the presence of IL‐8 (100 ng/mL) with or without siRNA of Twist. Magnification: 100×; Scale bars: 100 μm. F, Histogram of average numbers of penetrated cells per microscopic field. *P < 0.05

We examined whether IL‐8 induced alterations of EMT in PDAC cells. To this end, qRT‐PCR was performed to determine several common transcription regulators of EMT after IL‐8 treatment for 24 hours, and only the expression level of Twist increased by approximately ninefold for PANC‐1 and 38‐fold for BxPC‐3, respectively (Figure 5A). To further support the PCR‐based data on Twist leading EMT, Western blotting analysis revealed Twist expression was indeed upregulated resulting in repression of the epithelial marker E‐cadherin and induction of the mesenchymal marker vimentin (Figure 5B). In addition, metastatic lesions derived from the IL‐8 group showed higher expression of Twist than PBS both in the lung and liver lesions (Figure 5C and Figure S2). Then, to demonstrate the induction of EMT phenotype by IL‐8 was regulated by increased Twist expression, RNA interference was used to deplete Twist expression in PANC‐1 and BxPC‐3 cells, and the EMT features were examined in the presence of IL‐8 using Western blotting. As shown in Figure 5D, IL‐8‐induced traits were attenuated with knockdown Twist expression, suggesting a Twist‐dependent manner. In line with Western blotting data, the enhancement of migration and invasion caused by exogenous IL‐8 was abolished by suppressing Twist expression as well (Figure 5E,F and Figure S3). Furthermore, as demonstrated in Figure S4, Twist expression level in PDAC cells (PANC‐1 and BxPC‐3) was much higher than normal pancreatic ductal epithelial cell line (hTERT‐HPNE). Altogether, these results affirmed that IL‐8 promotion of cellular motility in PDAC depended on Twist upregulation.

### 3.5 The expression level of IL‐8 in tumor stroma was closely correlated with clinicopathological parameters and patients prognosis

**Figure 6:**
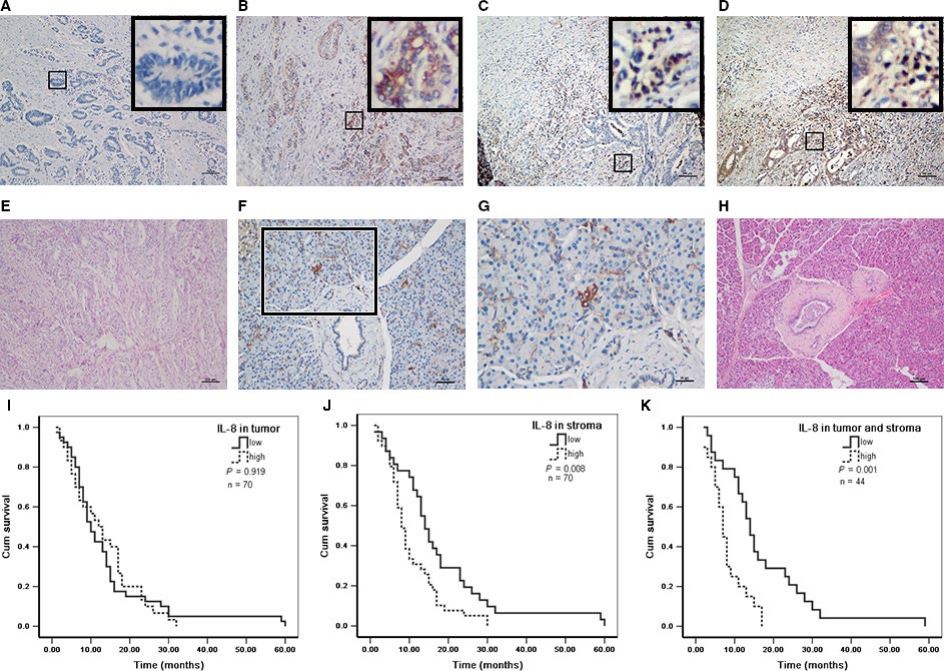
High IL‐8 expression level in tumor stroma correlated with shorter overall survival in patients with pancreatic ductal adenocarcinoma (PDAC). A‐H, Representative images of IL‐8 immunohistochemical staining in PDAC tissues and normal pancreas. A, Low expression of IL‐8 both in cancer cells and tumor stroma; B, High expression of IL‐8 in cancer cells and low expression in tumor stroma; C, Low expression of IL‐8 in cancer cells and high expression in tumor stroma; D, High expression of IL‐8 both in cancer cells and tumor stroma; E, H&E staining of PDAC tissues; F, Low expression of IL‐8 in normal pancreas; G, Magnified image of (F). Several acinar cells showed strong expression of IL‐8 in cytoplasm; H, H&E staining of normal pancreas tissue. Magnification: 100× (A‐F and H) and 400× (G); Scale bars: 100 μm (A‐F and H) and 25 μm (G). I‐K, The patients were divided into low and high group according to the immunohistochemical score (IHS) of IL‐8. IHS scores of 0‐4 were regarded as low expression and 5‐12 as high expression. Kaplan‐Meier analysis of overall survival in PDAC was performed between the two groups. I, IL‐8 expression in cancer cells (high vs low); J, IL‐8 expression in tumor stroma (high vs low); K, IL‐8 expression both in cancer cells and tumor stroma (high vs low)

Although IL‐8 expression in PDAC tissues has been estimated before,^23^ its information is scarce. Whereupon, we were interested in evaluating the expression and clinical significance of IL‐8 in PDAC biopsies. First, immunohistochemistry was performed in tissue samples from 70 patients with PDAC and 10 patients as control, and then, we found that IL‐8 protein was primarily observed in the cytoplasm of cancer cells and scattered distribution in tumor stroma (Figure 6A‐E). The immunoreactive signals were calculated based on previous study reported,^17^ in which immunohistochemical score (IHS) of 0‐4 was regarded as low expression and 5‐12 as high expression. In PDAC tissues, low‐level IL‐8 expression was 40 specimens (57.1%) in cancer cells and 31 specimens (44.3%) in tumor stroma, meanwhile, 30 in cancer cells and 39 in tumor stroma as high‐level expression. In contrast, there was no remarkable presence or very low expression of IL‐8 in pancreatic ductal epithelial cells and most of acinar cells in normal pancreas tissue. Intriguingly, a few of acinar cells were observed strong positive expression of IL‐8 in cell cytoplasm, suggesting some special cellular function (Figure 6F‐H). The relationship between clinicopathological variables and IL‐8 expression was shown in Table 1. As demonstrated in the table, high‐level IL‐8 expression in tumor stroma was correlated with high frequent lymph node metastasis, high density of tumor CD68 but not CD163 positive macrophages. However, there was no meaningful correlation between IL‐8 expression in cancer cells and the clinicopathological characteristics. To confirm the expression correlation between IL‐8 and CD68, we performed double immunofluorescent staining in PDAC tissue, and the result demonstrated that IL‐8 and CD68 were coexpressed in some cells, most likely TAMs (Figure S5). Kaplan‐Meier analysis was performed to evaluate the potency of IL‐8 expression on PDAC patients’ survival. It was found that significant correlation between shorter overall survival time and high‐level IL‐8 expression in tumor stroma or both tumor stroma and cancer cells, while no significant relationship between patient's survival and IL‐8 expression in cancer cells (Figure 6I‐K). Therefore, IL‐8 expression level in tumor stroma played an important role in PDAC lymph node metastasis and patient prognosis.

**Table 1:** Association between IL‐8 expression and clinicopathological characteristics in PDAC

Characteristics	n	IL‐8 expression in tumor			IL‐8 expression in stroma		
		Low	High	*P*	Low	High	*P*
Tumor size							
≤3.0 cm	22	11	11	0.414	10	12	0.894
>3.0 cm	48	29	19		21	27	
Histopathologic grade							
Grade 1	23	13	10	0.624	12	11	0.564
Grade 2	25	16	9		11	14	
Grade 3	22	11	11		8	14	
T stage							
T1/2	13	6	7	0.375	4	9	0.277
T3/4	57	34	23		27	30	
Lymph nodes status							
Negative	29	20	9	0.093	18	11	0.012^*^
Positive	41	20	21		13	28	
Distant metastasis							
Negative	58	34	24	0.583	26	32	0.841
Positive	12	6	6		5	7	
CD68^+^ macrophages							
Low	36	23	13	0.241	21	15	0.015^*^
High	34	17	17		10	24	
CD163^+^ macrophages							
Low	35	22	13	0.334	17	18	0.47
High	35	18	17		14	21	

a PDAC, pancreatic ductal adenocarcinoma.

*P < 0.05.

## 4 DISCUSSION

Numerous studies have demonstrated that TAMs resemble M2 macrophages associated with parasite clearance, wound healing, and dampen immune responses, while M1 macrophages are involved in the adaptive immune system.^24^ In the present study, we found that CM from PDAC cells was able to facilitate the recruitment of THP‐1 monocytes in vitro, and they were activated and differentiated toward tumor‐driven like macrophages, which seemed display a M2 phenotype. It has been reported that PDAC‐CM could induce a strong differentiation of PBMCs toward M2 like TAMs with high expression of migration‐stimulating factor (MSF),^12^ and this similar phenomenon is observed in the research of esophageal squamous cell carcinoma and breast cancer.^25^, ^26^ However, PDAC‐CM is also able to polarize PBMCs or U937 monocytes toward a mixed phenotype macrophages.^11^, ^27^ More interesting, Ole and his colleagues find that both M1 and M2 types of TAMs promote EMT process of PDAC cells.^9^ Hence, it is full of controversy with regard to the paradigm of TAMs due to the high plasticity of macrophages in the tumor microenvironment, and further studies are required to gain further insight.

Tumor‐associated macrophage‐derived cytokines display pro‐tumor activity through several mechanisms such as immune escape, extracellular matrix remodeling, angiogenesis, and tumor growth as well as cancer cells migration and invasion. Prior studies have verified several factors derived from TAMs important for tumor progression, including TGF‐β and VEGF.^28^ In our study, we identified the most abundant cytokine secreted by tumor‐driven like macrophages was IL‐8 which was capable to promote mobility of PDAC cells in vitro and in vivo. It has been also proven that TAMs‐derived IL‐8 plays a vital role in tumor progression in other malignancies.^18^, ^29^ Recently, a research using different TAMs model indicated TAMs‐derived IL‐10 enhanced the metastasis of PDAC cells.^10^ In that study, the authors chose mouse macrophages RAW 264.7 as the origin of TAMs following by treated with IL‐4, which are quite different from human monocyte cell lines THP‐1 used in this study. In addition, we further found that IL‐8 secreted by tumor‐driven like macrophages depended on activation of STAT3 but not NF‐κB signaling pathway. According to some reports, STAT3 is constitutively activated in cancer cells as well as tumor‐infiltrating immune cells, including TAMs,^30^ resulting in suppression of pro‐inflammatory cytokine and chemokine production. STAT3 activation is fundamental for macrophage differentiation into M2 phenotype,^31^ In consideration of the importance of STAT3 in TAMs, STAT3 signaling has increasingly become a potential new therapeutic strategies to target TAMs.^32^

Tumor EMT is a phenotypic switch through which epithelial tumor cells reduce cell polarity and cell‐to‐cell contacts, and convert into a mesenchymal phenotype, leading to enhanced tumor cell migration and invasion, increased metastatic propensity. IL‐8 has been demonstrated to involve in the EMT process which enhanced tumor cell migration and invasion.^33^ In our experiment, we found IL‐8 induced PDAC cells EMT switch with decreased expression of epithelial marker E‐cadherin and increased expression of mesenchymal marker vimentin. Previous coculture experiments have indicated that TAMs were capable to induce EMT in PDAC cells.^9^, ^10^ However, the exact responsible regulator is unknown. Then, our data from both qRT‐PCR and Western blotting confirmed that Twist played a key role in IL‐8 induced EMT of PDAC cells. Recent studies^34^ make it clear that Twist expression is associated with tumor aggressiveness and metastatic potential, especially function as a key regulator of EMT. In PDAC, Twist expression level is significantly higher in cancerous tissues compared with non‐cancerous tissues, and it is revealed to involve in tumor progression.^35^, ^36^ Taken together, our results illustrated the potential molecular mechanism of Twist expression in PDAC by cytokines derived from TAMs.

To evaluate the potential clinical relevance of our findings, the presence of IL‐8 expression was determined in PDAC samples using immunohistochemistry. The results showed that both cancer cells and tumor stroma were positive expression of IL‐8, while normal pancreas tissue barely expressed, which is in accord with previous reports.^23^ IL‐8 expression level in tumor stroma but not cancer cells was correlated with lymph nodes metastasis. More importantly, there was also a significant correlation between IL‐8 expression level and the number of tumor CD68 positive but not CD163 positive macrophages, and we also found that IL‐8 and CD68 were coexpressed in PDAC tissue. Collectively, these data suggested that IL‐8 was possibly present of autocrine and paracrine effects on PDAC cells. Since IL‐8 has not been characterized prognostic value in PDAC before, we performed survival analysis by the Kaplan‐Meier method. IL‐8 high expression level in tumor stroma but not cancer cells, and in both cancer cells and tumor stroma, was negatively correlated with patient outcome. And what is noteworthy is that pancreatic cancer cells are not the only source of IL‐8 in the tumor inflammatory microenvironment. Multiple cell types in the tumor microenvironment, such as neutrophils, macrophages, or fibroblasts, can secrete IL‐8 in response to kinds of stress factors.^37^ Conversely, high‐level of IL‐8 expression can also lead to intensive recruitment of neutrophils and TAMs.^38^ Recently, Fang et al^39^ have showed that higher levels of IL‐8‐positive Tumor‐infiltrating inflammatory cells (TIICs) but not tumor cells in PDAC patients correlated with worse prognosis, and as we know, TAMs is one of the most important TIICs subtype. In addition, many of experimental studies have characterized that there was higher serum level of IL‐8 in patients with PDAC compared to healthy controls, suggesting the diagnostic potential as novel clinical marker of PDAC patients.^40^, ^41^

In conclusion, the present study represented a reciprocal relationship between PDAC cells and tumor‐driven like macrophages. As a multifunction chemokine, IL‐8 in our study was just investigated its effect on EMT switch in PDAC cells, and hence further exploration will certainly be necessary to figure out its other effect on cancer stem cell, angiogenesis, and chemoresistance. In a word, blocking IL‐8 signaling within the tumor microenvironment will probably provide therapeutic benefit for PDAC.
