# Supplementary figures and images for: Tumor‐driven like macrophages induced by conditioned media from pancreatic ductal adenocarcinoma promote tumor metastasis via secreting IL‐8
Source: Cancer Med. 2018 Oct 12;7(11):5679–90. doi: 10.1002/cam4.1824 (PMC6246928; doi:10.1002/cam4.1824)

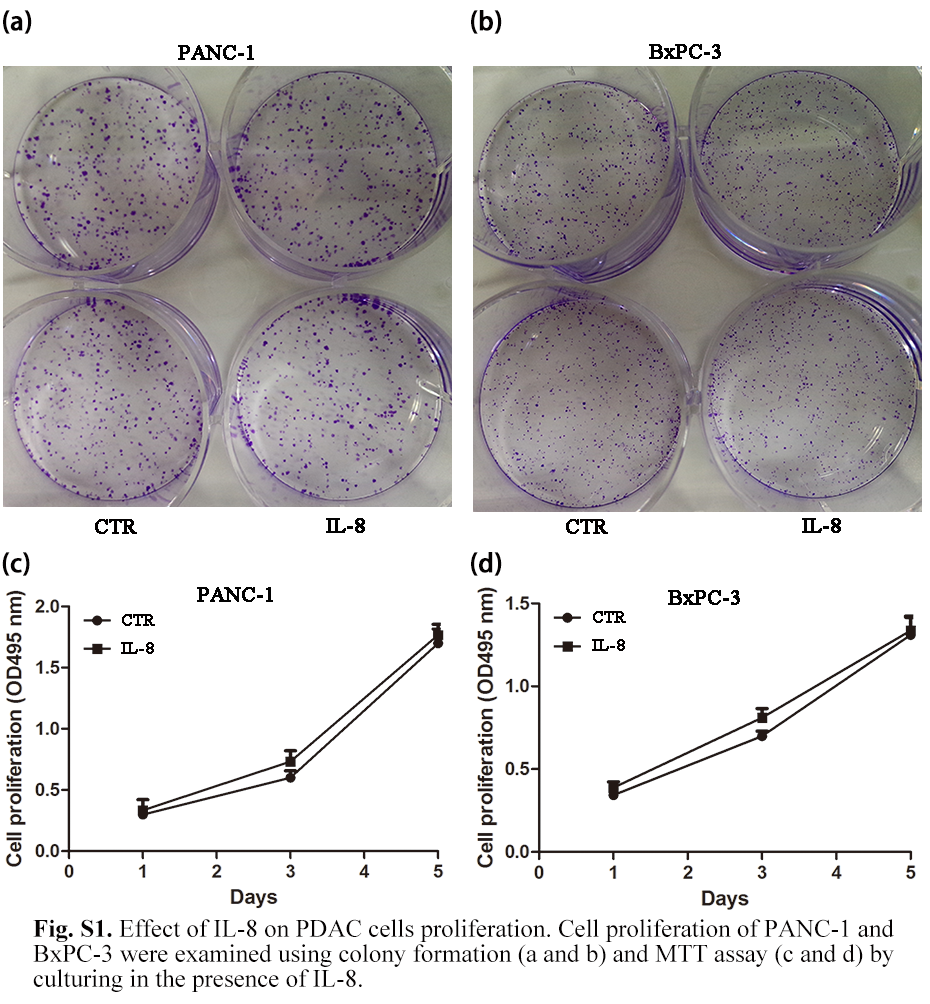

Supplement: Supplementary file 1 [file CAM4-7-5679-s001.tif]

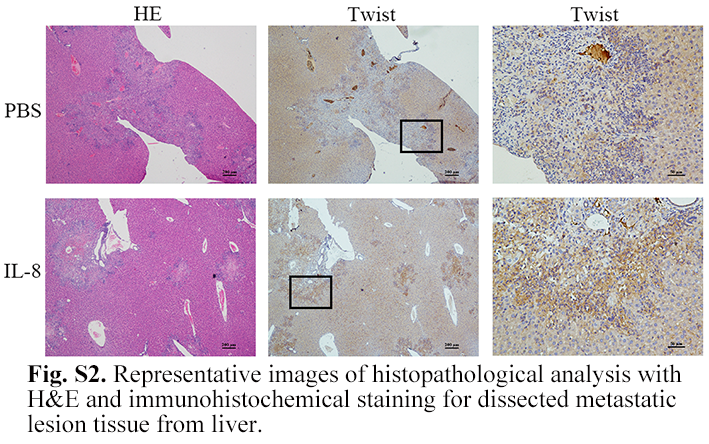

Supplement: Supplementary file 2 [file CAM4-7-5679-s002.tif]

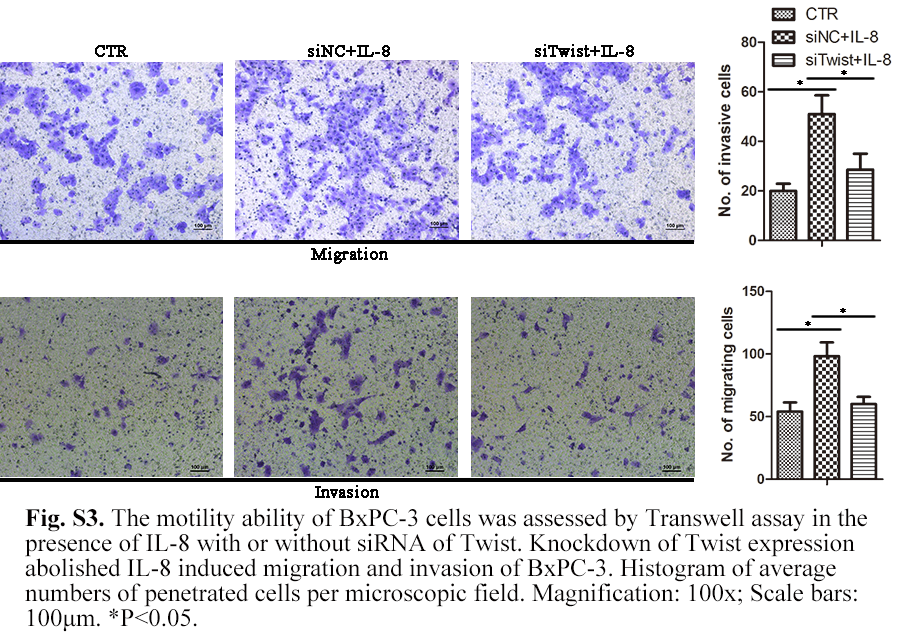

Supplement: Supplementary file 3 [file CAM4-7-5679-s003.tif]

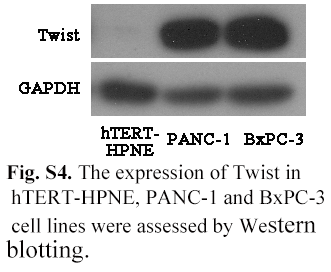

Supplement: Supplementary file 4 [file CAM4-7-5679-s004.tif]

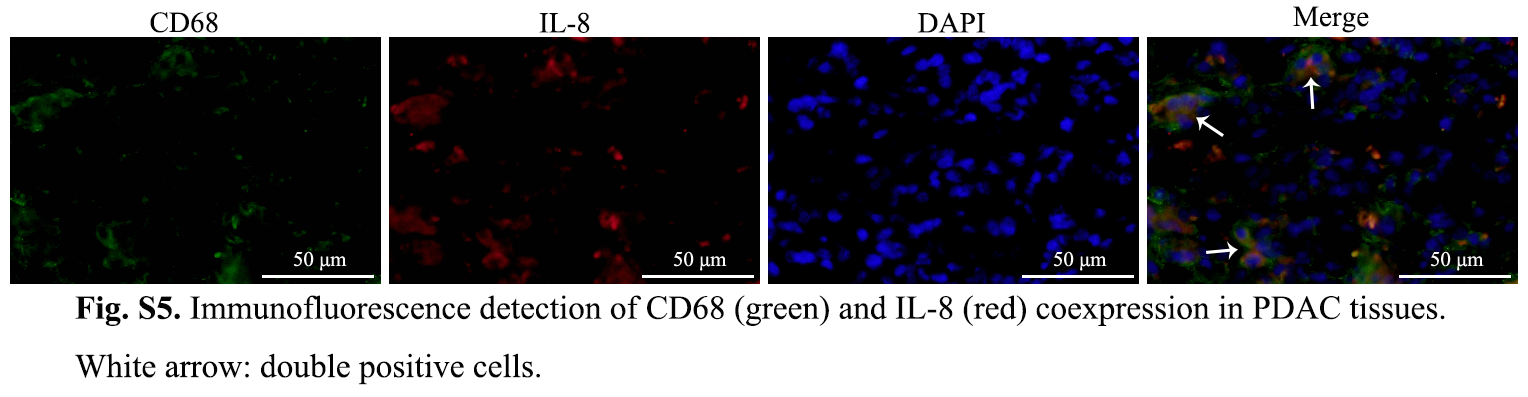

Supplement: Supplementary file 5 [file CAM4-7-5679-s005.tif]
