# Supplementary material for: Tumor‐driven like macrophages induced by conditioned media from pancreatic ductal adenocarcinoma promote tumor metastasis via secreting IL‐8
Source: Cancer Med. 2018 Oct 12;7(11):5679–90. doi: 10.1002/cam4.1824 (PMC6246928; doi:10.1002/cam4.1824)
Supplement: Supplementary file 6 [file CAM4-7-5679-s006.docx]

**Supporting information**

**Western blotting**

Total cell lysates were harvested in RIPA buffer (Beyotime, Shanghai, China) with protease inhibitors. Equal amounts of total protein were separated in 10% SDS-polyacrylamide mini-gels. After electrophoresis, the protein were transferred to polyvinylidene difluoride (PVDF) membranes, and blocked with 5% non-fat dry milk in 0.1% Tween 20/Tris-HCI-buffered saline (TBST, pH 7.4) for 1 h at room temperature. Finally, the protein membranes were incubated with corresponding primary antibody and respective horseradish peroxidase (HRP)-conjugated secondary antibody sequentially. Signals were detected by enhanced chemiluminescence. GAPDH was used as the housekeeping gene control. The primary antibodies were used as follows: anti-phospho-STAT3, 1:1,000 (Cell Signaling Technology, Danvers, MA, USA); anti-STAT3, 1:1,000 (Cell Signaling Technology); anti-phospho-NF-κB,1:1,000 (Cell Signaling Technology); anti-NF-κB,1:1,000 (Cell Signaling Technology); anti-E-cadherin, 1:1,000 (Cell Signaling Technology); anti-Vimentin, 1:1,000 (Cell Signaling Technology); anti-Twist, 1:1,000 (Abcam, Cambridge, UK); and anti-GAPDH, 1:1,000 (Cell Signaling Technology).

**Monocyte chemotaxis and adhesion assay**

Chemotaxis assays were conducted in 24-well modified Boyden chamber (Corning Life Sciences, Corning, MA, USA) as previously described[^42^](#_ENREF_42). Briefly, THP-1 monocytes were washed and resuspended in fresh RPMI 1640. Then, 100 µL of cell suspension (1 × 10^6^/mL) was loaded into insert of the Boyden chamber, and the lower well contained either control media or indicating CM. After allowing migrating for 1 h at 37 ℃ in an atmosphere of 5% CO_2_, migrated THP-1 monocytes were fixed with methanol and stained with Crystal Violet dye. The non-migratory cells were removed with cotton swabs, and the number of migratory cells were observed and counted under a microscope at 400× magnification.

Adhesion assays were performed in 96-well plate. At first, the plate was pre-coated with 40 µg/ml Matrigel (BD Biosciences, Bedford, MA, USA) into the bottom and incubated in the room temperature overnight. On the second day, THP-1 monocytes (1× 10^5^/100 µL) were suspended in indicating CM and seeded into the pre-treated plate. After incubating at 37℃/5% CO_2_ for 1 h, the non-adhered cells were removed by gentle washing PBS three times, and the number of adhered cells were observed and counted under a microscope.

**Transwell migration and invasion assays**

Cell migration and invasion was estimated using 24-well transwell chambers polycarbonate filter of 8 µm pore size (Corning Life Sciences). For invasion assay, chambers were pre-coated with 250 µg/ml Matrigel (BD Biosciences) to the upper surface of each filter. PANC-1 and BxPC-3 cells (2× 10^4^/well) treated with 100 ng/ml IL-8 (Pepro Tech Inc.) or corresponding CM with/without transfecting siRNA targeted Twist gene were loaded into an insert and 500 µl standard medium with 5% fetal bovine serum was added to the bottom chamber. Chambers were incubated in an atmosphere of 5% CO_2_ at 37℃for 15 h in the migration assay and 36 h in the invasion assay. Following incubation, cells on the upper surface of each filter were wiped off with a cotton swab. Cells on the lower surface of filter were then fixed with methanol, stained with 0.1% crystal violet, washed with water and air-dried. For each filter, the number of migrated or invaded cells in five random fields at 200×magnifications was counted. Data were presented as mean ± SD.

**qRT-PCR analysis**

Total RNA was extracted from different cells by Trizol reagent (Invitrogen, Carlsbad, CA, USA) and cDNA was synthesized with PrimeScript RT kit (Takara, Dalian, China) following the manufacturer’s instructions. The resulting cDNA was used for quantitative real-time PCR with SYSB PrimeScript RT-PCR kit (Takara) using LightCycler480 Realtime PCR System. The relative transcript level of each gene was obtained by the 2^-ΔΔCt^ method and normalized with respect to the housekeeping gene GAPDH. All specific primers are listed in Table S1.

**Table S1.** Primer Sequences for qRT-PCR.

| Gene | Name | Sequence |
| --- | --- | --- |
| Cytokines | | |
| TNF-α | Forward primer | 5’- TCTCGAACCCCCGAGTGACA -3’ |
|  | Reverse primer | 5’- GGCCCGGCGGTTCA -3’ |
| IL-6 | Forward primer | 5’- AATAACCACCCCTGACCCAAC -3’ |
|  | Reverse primer | 5’- ACATTTGCCGAAGAGCCCT -3’ |
| IL-10 | Forward primer | 5’- AACAAGAGCAAGGCCGTGG -3’ |
|  | Reverse primer | 5’- GAAGATGTCAAACTCACTCATGGC -3’ |
| VEGF | Forward primer | 5’- ATGACGAGGGCCTGGAGTGTG -3’ |
|  | Reverse primer | 5’- CCTATGTGCTGGCCTTGGTGAG -3’ |
| IL-8 | Forward primer | 5’- AAACCACCGGAAGGAACCAT -3’ |
|  | Reverse primer | 5’- CCTTCACACAGAGCTGCAGAAA -3’ |
| CXCL-12 | Forward primer | 5’- CCCGAAGCTAAAGTGGATTC -3’ |
|  | Reverse primer | 5’- TTCAGAGCTGGGCTCCTACT -3’ |
| CCL-18 | Forward primer | 5’- CTCTGCTGCCTCGTCTATACCT -3’ |
|  | Reverse primer | 5’- CTTGGTTAGGAGGATGACACCT -3’ |
| IL-1α | Forward primer | 5’- TCTGTTCTTGGGAATCCATGG -3’ |
|  | Reverse primer | 5’- TCAGTGATGTTAACTGCCTCCAG -3’ |
| TGF-β | Forward primer | 5’- AAGGACCTCGGCTGGAAGTGC -3’ |
|  | Reverse primer | 5’- CCGGGTTATGCTGGTTGTA -3’ |
| MMP-2 | Forward primer | 5’- GCAAGTCCCGTTCCGCTTCC -3’ |
|  | Reverse primer | 5’- CAGTACCAGTGTCAGTATCAGC -3’ |
| MMP-9 | Forward primer | 5’- CGGAGTGAGTTGAACCAG -3’ |
|  | Reverse primer | 5’- GTCCCAGTGGGGATTTAC -3’ |
| CCL-2 | Forward primer | 5’- AGAATCACCAGCAGCAAGTGTCC -3’ |
|  | Reverse primer | 5’- TCCTGAACCCACTTCTGCTTGG -3’ |
| The markers of macrophage | | |
| CD68 | Forward primer | 5’- GACCCACGACTGCCACTC -3’  -3’ |
|  | Reverse primer | 5’- GTGCTGTTGCTTGTTGGATG -3’ |
| CD163 | Forward primer | 5’- CGAGTTAACGCCAGTAAGG -3’ |
|  | Reverse primer | 5’- GAACATGTCACGCCAGC -3’ |
| CD204 | Forward primer | 5’- CCAGGGACATGGGAATGCAA -3’ |
|  | Reverse primer | 5’- CCAGTGGGACCTCGATCTCC -3’ |
| HLA-DR | Forward primer | 5’- TGTAAGGCACATGGAGGTGA -3’ |
|  | Reverse primer | 5’- ATAGGGCTGGAAAATGCTGA -3’ |
| EMT regulatory factors | | |
| Snail | Forward primer | 5’- CTTCCAGCAGCCCTACGA -3’ |
|  | Reverse primer | 5’- AGCCTTTCCCACTGTCCTC -3’ |
| Slug | Forward primer | 5’- TTTCTGGGCTGGCCAAACATAAGC -3’ |
|  | Reverse primer | 5’- ACACAAGGTAATGTGTGGGTCCGA -3’ |
| Twist | Forward primer | 5’- AGTCTTACGAGGAGCTGCAGACG -3’ |
|  | Reverse primer | 5’- AGGAAGTCGATGTACCTGGCCG -3’ |
| GAPDH | Forward primer | 5’- GCACCGTCAAGGCTGAGAAC -3’ |
|  | Reverse primer | 5’- TGGTGAAGACGCCAGTGGA -3’ |
